# Prevalence and risk of hepatitis E virus infection in the HIV population of Nepal

**DOI:** 10.1186/s12985-017-0899-x

**Published:** 2017-11-21

**Authors:** Ananta Shrestha, Anurag Adhikari, Manjula Bhattarai, Ramanuj Rauniyar, Jose D. Debes, André Boonstra, Thupten K. Lama, Mamun Al Mahtab, Amna Subhan Butt, Sheikh Mohammad Fazle Akbar, Nirmal Aryal, Sapana Karn, Krishna Das Manandhar, Birendra Prasad Gupta

**Affiliations:** 1 Liver Foundation Nepal, Kathmandu, Nepal; 2 Kathmandu Research Institute for Biological Sciences, Lalitpur, Nepal; 3 Department of Medicine, University of Minnesota, Minneapolis, MN USA; 4 Department of Gastroenterology and Hepatology, Erasmus MC, Rotterdam, the Netherlands; 5 Civil Service Hospital of Nepal, Kathmandu, Nepal; 6 Department of Hepatology, BangabandhuShiekhMujib Medical University, Dhaka, Bangladesh; 7 Department of Medicine, The Aga Khan University, Karachi, Pakistan; 8 Toshiba General Hospital, Tokyo, Japan; 9 Virology Unit, Central Department of Biotechnology, Tribhuvan University, Kathmandu, Nepal; 10 St. Xavier’s College Maitighar, Kathmandu, Nepal

**Keywords:** HIV, Blood donor, HEV, Kathmandu, Immunocompromised

## Abstract

**Background:**

Infection with the hepatitis E virus (HEV) can cause acute hepatitis in endemic areas in immune-competent hosts, as well as chronic infection in immune-compromised subjects in non-endemic areas. Most studies assessing HEV infection in HIV-infected populations have been performed in developed countries that are usually affected by HEV genotype 3. The objective of this study is to measure the prevalence and risk of acquiring HEV among HIV-infected individuals in Nepal.

**Methods:**

We prospectively evaluated 459 Human Immunodeficiency Virus (HIV)-positive individuals from Nepal, an endemic country for HEV, for seroprevalence of HEV and assessed risk factors associated with HEV infection. All individuals were on antiretroviral therapy and healthy blood donors were used as controls.

**Results:**

We found a high prevalence of HEV IgG (39.4%) and HEV IgM (15.3%) in HIV-positive subjects when compared to healthy HIV-negative controls: 9.5% and 4.4%, respectively (OR: 6.17, 95% CI 4.42–8.61, *p* < 0.001 and OR: 3.7, 95% CI 2.35–5.92, p < 0.001, respectively). Individuals residing in the Kathmandu area showed a significantly higher HEV IgG seroprevalance compared to individuals residing outside of Kathmandu (76.8% vs 11.1%, OR: 30.33, 95% CI 18.02–51.04, *p* = 0.001). Mean CD4 counts, HIV viral load and presence of hepatitis B surface antigen correlated with higher HEV IgM rate, while presence of hepatitis C antibody correlated with higher rate of HEV IgG in serum. Overall, individuals with HEV IgM positivity had higher levels of alanine aminotransferase (ALT) than IgM negative subjects, suggesting active acute infection. However, no specific symptoms for hepatitis were identified.

**Conclusions:**

HIV-positive subjects living in Kathmandu are at higher risk of acquiring HEV infection as compared to the general population and to HIV-positive subjects living outside Kathmandu.

## Background

Infection with the hepatitis E virus (HEV) is the most frequent cause of acute hepatitis among adults in developing countries with poor sanitation and hygiene [1, 2]. Four genotypes of the virus have been identified with different epidemiological and clinical characteristics and are capable of infecting and causing disease in human [3]. HEV genotype 1 and 2 are restricted to human and is associated with consumption of contaminated water, which consequently leads to occasional outbreaks or sporadic acute hepatitis cases in developing countries. HEV genotypes 3 and 4 are considered zoonotic and transmission is related to consumption of raw meat products or farming. These genotypes cause sporadic acute hepatitis in industrialized nations and occasionally can progress into a chronic infection in immunosuppressed hosts [4, 5]. Nepal is an endemic region for HEV, particularly genotype 1, and repeated epidemic outbreaks have been documented since 1973 [6, 7]. Sporadic cases of acute hepatitis E occur in-between these major epidemics and account for 50% of acute hepatitis cases in the country [8].

The clinical course and prevalence of HEV infection in subjects infected with human immunodeficiency virus (HIV) in endemic regions has not been well established. Conflicting reports exist regarding the susceptibility to HEV infection in HIV-positive subjects from different areas of world [5, 9, 10]. Similarly, the course and severity of HEV infection in HIV-infected subjects is a matter of debate: while some studies report unrecognized self-limiting disease, others report chronic infection and rapid progression to cirrhosis [11, 12]. Nepal has nearly 40,000 people living with HIV making a prevalence rate of 0.2% amongst adults [13]. HIV-infected persons tend to be socially marginalized and often have poor access to acceptable sanitary standards, therefore possibly being at higher risk for HEV infection. In this study, we addressed HEV infection in a cohort of HIV-infected subjects enrolled in the antiretroviral treatment program of Nepal. We explored risk factors for HEV infection in HIV-positive subjects and the relationship between immune parameters and characteristics of HEV infection.

## Methods

### Study subjects

This study was conducted among HIV-positive subjects from six districts of Nepal (Kathmandu, Lalitpur, Kaski, Sunsari, Bharatpur and Parsa) attending their respective antiretroviral therapy (ART) centers under the National ART program of Nepal, between January 2015 to March 2015. Six hundred and thirteen subjects visited the center during the study period; out of which 459 subjects were 18 years or older, and consented to participate in the study. A single time-point assessment and blood sample collection was done to obtain demographic, virological, clinical and biochemical data. Demographic and clinical data were recorded in a predesigned questionnaire. Patients with antibodies against the hepatitis C virus (HCV) were considered as co-infected irrespective of whether HCV RNA amplification was performed. A preliminary letter with some of the preliminary findings pertaining to HEV IgM was recently published by our group [14].

As controls, we used blood samples and demographic data of 581 healthy voluntary blood donors aged ≥18 years from the central blood bank, Kathmandu, Nepal. These blood samples were collected between February and March 2014 after informed consent for our another study to address HEV in healthy donors, and has been described in detail before [15]. The control group constituted 401 male and 180 females. Among the screened population, 37 were from Kathmandu valley and 544 were from outside the valley. Written consent was received from all participants and the study was approved by the Nepal Health Research Council ethical review board.

### Laboratory analysis

Blood was collected in EDTA tubes (Becton Dickinson, USA) and CD4 counts were determined using the FACS Count system (Becton Dickinson, USA) within 3 h of collection. Similarly, samples collected in Gel clot vials were centrifuged and sera were transferred to eppendorf tubes and stored at −20° Celsius. Biochemical assays for Alanine aminotransferase (ALT), Aspartate aminotransferase (AST) and Gamma glutamyltranspeptidase (GGT) were done using Bio-Slide Technology (J&J, USA). The serology for anti-HEV IgM and anti-HEV IgG was performed using ELISA (Wantai Biological Pharmacy Enterprise, China). ELISA was undertaken in accordance with the manufacturer’s instructions. Samples with anti-HEV sample rate/cutoff rate (S/CO) ratios >1 were considered seropositive. HBsAg was assayed using Abbott-AuszymeMc (Abbott Laboratories, USA) and anti-HCV was performed by HCV ELISA (Ortho Diagnostic Systems). HIV RNA was isolated using the Nucleospin viral RNA isolation kit (MACHEREY-NAGEL, Germany) according to the manufacturer’s instruction. For viral RNA amplification and cDNA preparation artus HI Virus-1 QS-RGQ Kit (QIAGEN, Germany) was used and primers were supplied by the manufacturer as complete master mix.

The standard protocol for HEV RNA assay was adapted from previously described study by Jothikumar et al. [16]. HEV RNA was purified from 50 μL of serum and the GenMagScript One-Step RT-PCR Kit was used for detection. The 25 μL reaction mixture contained 12.5 μL of 2X One-Step RT-PCR reaction buffer (Genmagbio, Beijing, China), 0.2 μL of GenMagScript RT Enzyme Mix, 5 μL of RNA, and primers (forward primer: BDHEVF (5′-GGTGGTT TCTGGGGTGAC-3′) and reverse primer: BDHEVR (5′-A GGGGTTGGTTGGATGAA-3′) and probe (BDHEVP; 5′-TGATTCTCAGCCCTTCGC-3′) at concentrations of 200 nM. Additionally, internal controls, supplied in the kit (GenMagScript, China), were used to identify possible PCR inhibition as detected by fluorescence channel orange in Rotor gene Q. As an exogenous internal control sequence, lambda gene PCR product (278-bp) was added to the reaction mixture.

### Statistical analyses

Data was entered and analyzed using SPSS software version 23.0. Frequencies were calculated for categorical variables and mean ± SD were calculated for quantitative variables. *P* values of <0.05 were considered significant, unless described otherwise. Odds ratios (OR) with 95% confidence intervals were calculated. Categorical variables were compared using the chi-squared test, while the T test was used for comparing continuous variables. Spearman’s correlation coefficient was used to determine the degree of correlation between continuous variables.

## Results

**Fig. 1:**
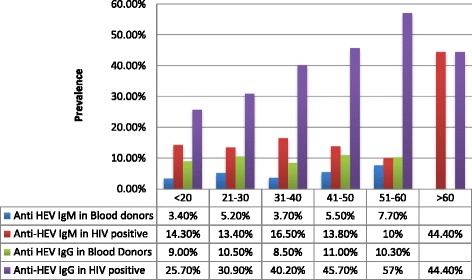
Age-specific prevalence of anti-HEV IgM and anti-HEV IgG in control subjects and HIV-infected subjects. Anti HEV IgM: blood donors versus HIV-positive individuals: age < 20 (*p* = 0.04); age 21–30 (*p* = 0.021); age 31–40 (*p* = 0.00); age 41–50 (*p* = 0.21); age 51–60 (*p* = 1.0). Anti HEV IgG: blood donors versus HIV-positive individuals: age < 20 (p = 0.021); age 21–30 (p = 0.00); age 31–40 (p = 0.00); age 41–50 (p = 0.00); age 51–60 (p = 0.00)

Four hundred and fifty-nine HIV-positive subjects and 581 blood donors (controls) were analyzed. Their demographic profiles are presented in Table 1. Seventy of 459 (15.3%) HIV-positive subjects were reactive for anti-HEV IgM as compared to 4.4% in the HIV-negative control (blood donor) group [*p* < 0.001; OR: 3.7 (2.35–5.92)]. Anti-HEV IgG was positive in 181 (39.4%) of the HIV-positive subjects as compared to 56 (9.5%) of blood donors [p < 0.001; OR: 6.17 (4.42–8.61)]. Age specific prevalence rates of both anti-HEV IgM and IgG were significantly higher in HIV-infected subjects as compared to control groups for individuals younger than 20 years, between 20 and 30 years and between 31 and 40 years of age (Fig. 1). Moreover, for anti-HEV IgG, but not IgM, the prevalence rates were also significant between 41 and 50 and 51–60 years of age (Fig. 1).

**Table 1:** Characteristics of study subjects and comparison between anti-HEV IgM positive and IgM negative group

Characteristics/ Variables	Total HIV+ subjects (*N* = 459) N (%) or mean ± SD	Anti-HEV IgM+(*N* = 70) N (%) or mean ± SD	Anti-HEV IgM–(*N* = 389) N (%) or mean ± SD	*P* value^b^
Age	36.21 ± 10.35	37.41 ± 11.02	35.99 ± 10.19	0.318
Gender (Male)	274 (59.69)	40 (57.14)	234 (60.15)	0.510^c^
Duration of HIV Diagnosis (years)	4.12 ± 2.57	4.57 ± 2.6	4.04 ± 2.56	0.081^d^
Living in Kathmandu Valley	198 (43.13)	57 (81.42)	141 (36.24)	<0.001^c,a^
CD4+ count (cells/μL)	301.97 ± 117.75	351.73 ± 109.78	293.02 ± 117.03	<0.001^a^
ALT	32 ± 12.39	53.9 ± 19.80	28.75 ± 3.79	<0.001^a^
AST	35 ± 8.15	42.77 ± 14.85	33.60 ± 5.13	<0.001^a^
HBsAg (n)	24 (5.2)	10 (14.28)	14 (3.59)	<0.001^c,a^
Anti HCV (n)	342 (74.50)	27 (38.57)	315 (80.97)	<0.001^c,a^
Anti-HEV IgG (n)	181 (39.43)	17 (24.28)	164 (42.15)	0.005^c, a^
HIV Viral load in log cps/ml	2.74 ± 0.46	2.90 ± 0.61	2.71 ± 0.44	0.016^a^

Abbreviations: CD4- Cluster of differentiations 4; ALT- Alanine aminotransferase; AST- Aspartate aminotransferase; GGT- Gamma glutamyltranspeptidase; HBsAg- Hepatitis B surface Antigen; HCV- Hepatitis C virus, HEV- Hepatitis E virus, HIV- Human Immunodeficiency virus

^a^Significant

^b^Significance of difference between anti-HEV IgM+ and IgM– group

^c^Chi squared test

^d^Mann Whitney U test

**Fig. 2:**
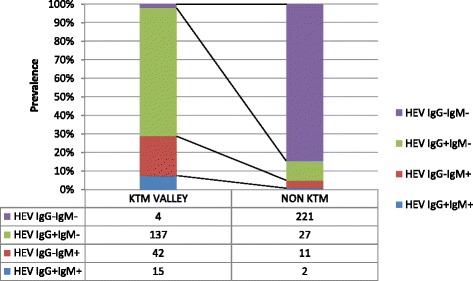
Prevalence of IgM and IgG anti-HEV in HIV-infected subjects from Kathmandu and outside Kathmandu Valley: The overall prevalence of IgM and IgG anti- HEV is higher in Kathmandu valley indicating that HIV-infected individuals living in Kathmandu are immune to HEV

We noticed a significant difference in the prevalence of anti-HEV IgG and IgM among HIV-positive study subjects living in Kathmandu, the capital of Nepal, and those residing outside of Kathmandu (Fig. 2). Overall anti-HEV IgG prevalence was 76.8% in HIV-positive subjects living in Kathmandu valley as compared to 11.1% of subjects living outside Kathmandu [*p* = 0.001; OR: 30.33 (18.02–51.04)]. Similarly, anti-HEV IgM was detected in 28.8% of HIV-positive subjects living in Kathmandu Valley as compared to 4.9% of those living outside Kathmandu [p = 0.001; OR: 7.71(4.07–14.58)].

We performed HEV PCR on samples of all 70 HIV-positive individuals and 27 blood donors that were positive for anti-HEV IgM. Seventeen (24%) of the HIV-positive individuals and 9 (33%) of the controls showed positive HEV RNA levels. (Table 2). The age-adjusted prevalence rates of anti-HEV IgM and anti-HEV IgG in the control group of healthy blood donors, as well as HIV-positive subjects living within Kathmandu and those living outside of Kathmandu are shown in Table 3.

**Table 2:** Comparison of various combinations of anti HEV IgM and IgG among HIV positive subjects and Blood Donors

Anti HEV antibodies	IgM+/IgG+	IgM+/IgG-	IgM−/IgG+	IgM−/IgG-	HEV RNA+
Blood Donors	9(1.5%)	18(3.1)	47(8.1)	507(87.3)	9(1.5%)
HIV positive Subjects	17(3.7%)	53(11.5%)	164(35.7%)	225(49%)	17(3.7%)

**Table 3:** Crude and age-adjusted prevalence rates of anti-HEV IgM and anti-HEV IgG among control group, HIV-positive subjects living inside and outside Kathmandu

		HIV (total)	Blood donors	HIV (Kathmandu)	HIV (Outside Kathmandu)
Anti-HEV IgM	Crude prevalence rate	15.31	4.64	28.93	4.9
	Age adjusted prevalence rate	17.54	3.6	30.61	4.21
Anti-HEV IgG	Crude prevalence rate	39.60	9.63	77.8	23.6
	Age adjusted prevalence rate	35.02	8.3	65.89	20.84

Lower mean CD4 counts, higher HIV viral load (log copies), higher ALT and AST, presence of HBsAg, and living-inside-Kathmandu valley were significantly associated with a higher rate of anti-HEV IgM seropositivity, while presence of HCV antibody was significantly associated with HEV IgM seronegativity. In contrast, age, gender and duration of HIV infection were not significantly associated to anti-HEV IgM status (Table 1).

**Fig. 3:**
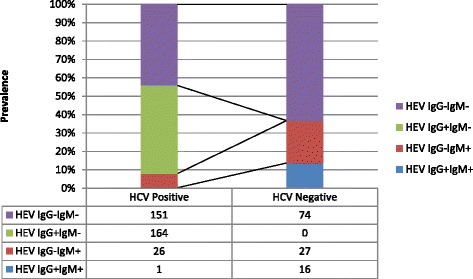
Prevalence of IgM and IgG anti-HEV in HIV-infected subjects with and without HCV co-infection: The prevalence of anti-HEV IgG in HCV co-infected subjects was 47.9% as compared 13.7% in those without co-infection [*p* < 0.001; OR: 5.88 (3.33–10.38)]

In multivariate analysis, living-inside-Kathmandu valley and anti-HCV negativity were independent risk factors associated with the presence of anti-HEV IgM positivity in HIV-infected subjects (Table 4). Interestingly, a CD4 count >200/μl, and a HIV viral load >3log copies/ml were also associated with presence of anti-HEV IgM. HIV viral load >3 log copies/ml, HCV co-infection and living-inside-the-Kathmandu valley were independent risk factors for anti-HEV IgG positivity (Table 4). The prevalence of anti-HEV IgG in HCV co-infected subjects was 47.9% as compared 13.7% in those without co-infection [*p* < 0.001; OR: 5.88 (3.33–10.38)] (Fig. 3).

**Table 4:** Multivariate analysis showing independent risk factors for anti-HEV IgM and anti-HEV IgG among HIV-positive subjects

Risk Factors	Odds Ratio for Anti-HEV IgM, 95% CI	*P* value^†^	Odds Ratio for Anti-HEV IgG, 95% CI	*P* value^γ^
CD4+ count >200cells/μl	5.109 [1.624–16.070]	0.005*	0.67 [.333–1.36]	0.273
Anti HCV Positive status	0.018 [0.006–0.056]	<0.001*	5.35 [2.44–11.69]	<0.001*
Residing in Kathmandu Valley	44.31 [14.507–135.353]	<0.001*	37.73 [20.98–67.87]	<0.001*
HIV viral load > 3log cps/ml	3.045 [1.25–7.39]	<0.001*	4.43 [1.67–11.73]	0.001*

CI- Confidence interval

*Significant ^†^association between risk factor and presence of anti-HEV IgM in serum of HIV-positive patients

^γ^association between risk factor and presence of anti-HEV IgG in serum of HIV-positive patients

The levels of the serum transaminases ALT and AST were also significantly higher in anti-HEV IgM positive subjects as compared to anti-HEV IgM negative HIV patients, suggesting clinically acute infection in these cases.

## Discussion

HEV infection is endemic in Nepal and cases occur in sporadic and epidemic fashion. The current study shows that HIV-infected individuals are at higher risk of acquiring acute HEV infection than healthy individuals in this area. Several studies from developed countries suggest that HEV is not more frequent in HIV-infected subjects compared to healthy controls [5, 10]. Kuniholm et al. reported that in the United States, HEV genotype 3 caused both acute and chronic HEV infection in HIV-positive subjects, but it was too rare to recommend routine surveillance, with only one chronic case in more than 300 tested individuals [5]. HEV genotype 3 infection is a food-borne infection and is related to consumption of uncooked meat products. Thus, in developed countries, distribution of HEV may be determined by eating habits and methods of processing pork meat [10]. In developing countries like Nepal, HEV is exclusively caused by genotype 1 infection [6–8, 17]. Contamination of the drinking water supply by HEV is common in developing nations and a large population is therefore exposed to the virus [6, 7, 18]. This likely explains the high prevalence of anti-HEV positivity in both HIV-infected as well as healthy controls in our study. However, whether HIV-infected subjects have a higher HEV attack rate as compared to the general population is not clear. HIV-infected subjects in Nepal are socially marginalized, and economically compromised [19]. Poor access to clean drinking water could possibly explain higher anti-HEV IgM prevalence in this group regardless of their immune status.

Our study showed that anti-HEV IgG seroprevalence was higher in HIV-infected subjects as compared to the control population. Similar observations were made in Russia, Argentina and Germany [20–22]. However, recent studies from the United Kingdom and Spain found that anti-HEV IgG prevalence in HIV-positive and –negative subjects were not different [10, 23].Further studies are needed to understand whether this discrepancy in findings is related to HEV genotype (HEV 1 vs 3), cultural habits of the geographical region or immune status.

Although the entire country of Nepal is listed as endemic region for HEV infection, most of the major epidemics have taken place in the Kathmandu Valley. Drinking water pipelines and sewage pipelines run underground close to each other in Kathmandu Valley. A breach in sewage pipelines and subsequent fecal contamination of the water supply leads to major epidemics. Such close proximity of drinking water supply and sewage pipelines are not seen in most of the rural areas of Nepal making them less prone to outbreaks of HEV. Previous studies have demonstrated that anti-HEV IgG prevalence was high in residents of Kathmandu valley and remarkably low in other areas of Nepal [7]. In the current study, age adjusted prevalence of anti-HEV IgG in HIV-positive subjects was 35% as compared to 8% in the control group. The prevalence of anti-HEV IgG in our study seems lower than that reported in a recent study by Izopet et al., which was conducted in a hospital of Kathmandu valley, which found 47.1% HEV seroprevalence in non-HIV-infected subjects [24]. When we segregated our study cohort on the basis of where the subjects live, the age adjusted prevalence rate of anti-HEV IgG among HIV-positive subjects from Kathmandu was 65.8%. This value was more in concordance with that in the general population of Kathmandu as reported by Izopet et al., but still higher in HIV-infected individuals compared to controls.

The control group in our study, composed of healthy blood donors, showed an anti-HEV IgG prevalence of 8.3%, which was in the same range as previously published data from Kathmandu [25, 26], but markedly lower than Izopet et al. [24]. This can be explained by the fact that 94% of the control group was formed by people living outside Kathmandu valley. Overall only 2% of the HIV-positive subjects living in Kathmandu lacked evidence of recent or remote infection (IgM or IgG positivity) as compared to 84.6% of subjects living outside Kathmandu. Recurrent epidemics in Kathmandu along with high prevalence of both anti-HEV IgM an IgG in subjects living in Kathmandu as compared to those living in other areas clearly indicate that HEV is largely confined to the Kathmandu valley.

In multivariate analysis, the current study identified independent risk factors for anti-HEV IgM and IgG positivity. While HIV viral load >3 log copies/ml and living in Kathmandu valley were common risk factors for anti-HEV IgM as well as IgG positivity, HCV co-infection was found to be a risk factor only for anti-HEV IgG positivity. Indeed, HCV co-infected subjects in our cohort showed a much higher evidence of immunity to HEV compared to HCV-negative (48% vs 13%). This is rather surprising since the predominant risk factor for HCV infection in Nepal is intravenous drug use. Whether intravenous drug use poses a risk for HEV exposure in HCV infected subjects leading to higher anti-HEV IgG prevalence is not known, and HEV is not known to be transmitted through blood, but it is a possibility that needs to be evaluated in future studies. Balyan et al. also reported high prevalence of anti-HEV IgG in Russian HIV-positive subjects and proposed common risk factors for transmission of both viruses [20]. Moreover, anti-HEV IgG positivity is higher in patients undergoing hemodialysis (which is subjected to frequent blood transfusions) with no clear explanation [27].

It has been reported that low CD4 counts in HIV-positive patients are associated with higher anti-HEV IgG seropositivity [23, 28]. Interestingly, we observed a higher rate of anti-HEV IgM positivity in subjects with CD4 counts >200. However, one should be cautious when interpreting the immune status (i.e. the immunoglobulin levels) in immunocompromised patients. A proportion, but not all, of those that tested positive for HEV IgM showed detectable levels of HEV RNA. This could be due to a) decrease in RNA levels before negativization of IgM in acute disease or b) lack of correlation between IgM and HEV viral load. The later situation has been described before and it is thought to be due to lack of international standardization of HEV primers for PCR [29].

HIV-positive subjects with acute HEV infection did not show marked ALT elevations as seen in immunocompetent individuals and none of them had bilirubin >2 mg/dl. Although anti-HEV IgM positive subjects had higher ALT levels than the remaining group, the degree of ALT elevation was less than 3 times the upper limit of normal and none showed symptoms of acute hepatitis. HEV is a non-cytopathic virus and the damage of hepatocytes is an immune mediated phenomenon. Therefore, the severity of hepatocyte injury in immunocompromised hosts might be limited.

## Conclusion

HIV-positive subjects living in Kathmandu are at higher risk of acquiring HEV infection as compared to the general population and to HIV-positive subjects living outside Kathmandu. Even though acute HEV infections are more frequently seen in HIV-positive subjects, the resulting health effects are not clear, as it does not seem to cause clinically evident disease. Further studies are necessary to understand the long-term implications of HEV infection in this population.

## Abbreviations

ALT: Alanine aminotransferase
AST: Aspartate aminotransferase
GGT: Gamma glutamyltranspeptidase
HBV: Hepatitis B Virus
HCV: Hepatitis C Virus
HEV: Hepatitis E virus
HIV: Human Immunodeficiency Virus
IDU: Intravenous drug user
